# Chicago Veterinary Society

**Published:** 1897-09

**Authors:** L. Campbell

**Affiliations:** Secretary


### CHICAGO VETERINARY SOCIETY.

July 8, 1897, owing to a quorum not being present, no meeting was held.

August 12, 1897, meeting called to order by President Walker. On motion, roll-call was dispensed with. Seventeen members were present. The minutes of the June meeting were read and approved. The application for membership of Dr. C. G. Gruner was presented, and after examination by the Board of Censors, Dr. Gruner was elected to membership.

The Treasurer's report showed $24.32 in the treasury. No report from the Secretary.

The Legislative Committee (Dr. Hughes, chairman) reported that the committee called upon Dr. Reynolds, of the City Health Department, in regard to establishing a meat- and milk-inspection in this city, which would be under the control of veterinarians. The committee was excellently received by Dr. Reynolds, who referred the committee to the Civil Service Commission. The latter, after hearing the desires of our committee, fell earnestly into our ideas, and promised that positions that should be filled by veterinarians should be filled by no one else, and also that the present quack city veterinarian to the Police Department would not continue to hold his position longer than for them to find an eligible practitioner. And further, that within three weeks an examination of members of this Society desiring city positions would be held for the purpose of finding competent men.

Moved by Dr. Henderson, seconded by Dr. E. L. Quitman, that the Secretary write the Civil Service Commissioners and ascertain the exact date of such examinations, and also sincerely thank them for their kindness in their reception of our committee and to our Society as a whole; voted and carried.

Motion by Dr. E. L. Quitman, seconded by Dr. Hawley, that this Society hold its November meeting on one of the days of the annual meeting of the Illinois State Veterinary Medical Association, and that the Literary and Publication Committee produce at least two papers for presentation to the Society on such occasion; voted and carried.

Motion by Dr. Robertson, seconded by Dr. Allen, to dispense with the regular order of business (no paper being presented), and to hear reports of unusual cases from any member present who may desire to state same.

Dr. E. L. Quitman related two cases to show the efficacy of potassium iodide and iodine in paralysis:

Case I.—Cocker spaniel, male, five or six years old; chronic constipation present. Paraplegia symptoms. These were so severe that the hindparts were dragged. A cathartic was given, with good responsive action, but no improvement of the paralysis. Strychnine in doses of one-ninetieth to one-thirtieth grain were given for two months; no improvement. Iodide of potassium and resublimed iodine were given, with great improvement in eight or nine days, and an entire cure in three weeks.

Case II.—Horse down in stall and unable to rise at first; later with aid stood up, but was unable to handle hindquarters properly. Strychnine was given, with no improvement. The case became worse. Would walk three or four steps and fall. Substituted the following: ℞.—Iod. pot., ʒj; iod. resub., gr. xv. Sig.—M. in sol., t. i. d.; and blistered the spine. Entire recovery in three weeks.

Moved by Dr. Hughes, seconded by Dr. Campbell, that the Society give a vote of thanks to Dr. Quitman; carried.

Dr. Hughes reported that the potassium-iodide treatment was recommended in the Royal College very highly, and that he had made successful treatment with it when strychnine had failed.

Dr. Robertson mentioned a case pending in court where a great controversy took place over the recovery of a supposedly stolen dog. Great diversity of opinion by experts was in evidence as to the dog's age (the age being conclusive as to ownership), and the Doctor wanted to know if any of the members present think the age of a dog can be determined by the teeth after the age of one year.

Dr. Hughes said that at the Royal Veterinary College students were expressly instructed not to try to tell the age by the teeth, as it was very risky and, in Dr. Hughes's opinion, a very doubtful proceeding; for his part, he would rather depend on the actions, antics, and appearance of the different breeds, rather than by the teeth.

Dr. Robertson held that in his opinion the age could not be told after one year, and never omits, when asked, to say that he does not know the age and cannot tell.

Dr. White stated that in his opinion dogs with even jaws can be approximately told after experience and careful study up to five years old.

Dr. Worms relies on the tartar and color of the teeth; he thinks more and more tartar and more yellowness, especially of the molars, occur year by year, and careful observation of this is some indication.

On motion, the regular order of business was recalled, and on motion the meeting adjourned.

L. CAMPBELL,
Secretary.

